# Commentary: “Prdm13 regulates subtype specification of retinal amacrine interneurons and modulates visual sensitivity”

**DOI:** 10.3389/fncel.2015.00424

**Published:** 2015-10-27

**Authors:** Hannah E. Bowrey, Morgan H. James

**Affiliations:** Brain Health Institute, Rutgers, The State University of New JerseyPiscataway, NJ, USA

**Keywords:** amacrine cell, subtype specification, Prdm13, retina, visual sensitivity, aliasing

The retina is a complex neural network whose primary role is to process raw visual images before conveying the results to the visual cortices of the brain. This is ultimately achieved by parallel populations of retinal ganglion cells, with considerable input from all other retinal cells, including photoreceptors, bipolar cells, horizontal cells, and amacrine cells (Gollisch and Meister, 2010). Thus, to fully understand the intricate diversity of retinal function, an understanding of these cells, and that of their subclasses, is critically important.

Amacrine cells are the most diverse class of retinal cells, having as many as 30 different subtypes (Masland, 2001). Such great variety of amacrine cells suggests many specific functions, some of which are well understood. For example, dopaminergic amacrine cells modulate retinal responsiveness under photopic and scotopic conditions (Witkovsky and Dearry, 1991), ultimately influencing center-surround balance of ganglion cells (Jensen and Daw, 1986), whereas starburst amacrine cells are important for direction selectivity (Yoshida et al., 2001). How amacrine cell subtypes are specified, however, and how those subtypes lead to broader visual functions, is relatively unknown.

In an elegant set of experiments, Watanabe et al. (2015) recently examined the role of Prdm13, a putative zinc-finger transcription factor, in the development of amacrine cells, and its associated role in visual sensitivity. Using retinas from developing (E11.5-P9) and mature (P21) mice, the authors demonstrated that in early development, Prdm13 was predominantly expressed in the inner nuclear layer (INL), the retinal layer in which most amacrine cells are localized, and demonstrated that Prdm13 is expressed in a particular subset of amacrine cells. Further, almost the entire population of Prdm13+ amacrine cells was either GABAergic or glycinergic, and most Prdm13+ amacrine cells expressed calbindin and calretinin (CALBs+), an indicator of amacrine cells containing nitric oxide, GABA, and/or substance P (Haverkamp and Wässle, 2000).

Forced expression of Prdm13 induced GABAergic and glycinergic amacrine cell types, suggesting a role for this transcriptional factor in the development of these cells. The authors also showed that *Prdm13*^−∕−^ mice exhibited reduced abundance of GABAergic and glycinergic amacrine cells, and reduced CALBs+ amacrine cells that project to the S2/S3 border of the inner plexiform layer (IPL: by ~30%). Together, these findings point to a role for Prdm13 in defining a subtype of GABAergic or glycinergic amacrine cells that project to the S2/S3 border bundle of the IPL.

Perhaps most interestingly, Watanabe et al. then probed the *in vivo* function of Prdm13+ retinal cells. Electroretinogram (ERG) analysis revealed no differences between wildtype (WT) and *Prdm13*^−∕−^ mice at 1 month of age. Scotopic and photopic ERG revealed typical a−, b−, and oscillatory potential-waveforms and amplitudes, indicating that WT and *Prdm13*^−∕−^ mice did not differ with respect to photoreceptor, bipolar, or amacrine cell function.

As a secondary *in vivo* functional assessment, the mice were subjected to an optokinetic response (OKR) assay, where head-fixed mice were exposed to moving sinusoidal gratings. Spatial frequency, temporal frequency, and contrast sensitivity were manipulated as eye-tracking movements of the mouse were calculated. Remarkably, in the majority of subjects, measurements of visual function in *Prdm13*^−∕−^ mice were shifted higher than those of WT mice, leading the authors to speculate that spatial and temporal frequencies and contrast sensitivities were enhanced in *Prdm13*^−∕−^ mice. This finding is particularly noteworthy: this is the first time a mutant mouse model has exhibited greater visual function than that of the WT mouse.

The findings of Watanabe et al. raise an important question. How can reducing the number of Prdm13+ amacrine cells lead to *greater* visual function? The authors suggest that because the CALBs+ S2/S3 border bundle was completely absent in the *Prdm13*^−∕−^ mouse retina, Type 2 amacrine cells may be Prdm13+. Type 2 amacrine cells synapse with Vglut3+ amacrine cells that contain glycine and glutamate (Knop et al., 2011). Additionally, Vglut3+ amacrine cells project to direction-sensitive ganglion cells, which play a role in directional selectivity associated with OKR (Lee et al., 2014). If Type 2 amacrine cells are Prdm13+, then Prdm13+ cells may negatively affect direction sensitive ganglion cell function via Vglut3+ amacrine cells, and the loss of Prdm13+ cells may lead to higher OKR sensitivities for *Prdm13*^−∕−^ mice. Thus, the authors suggest that this specific subset of amacrine cells may negatively modulate visual sensitivities. We find this to be a very reasonable explanation for these results.

Another possibility is that *Prdm13*^−∕−^ mice were able to detect stimuli of such high frequency as a result of aliasing, which occurs as a consequence of undersampling by the retina, leading to better than predicted visual performance. Aliasing is a common real-word phenomenon that has been demonstrated in several species including zebrafish (Maaswinkel and Li, 2003), guinea pigs (Bowrey et al., in press), and humans (Chui et al., 2005). A familiar example of this is when a wheel is spinning quickly in a clockwise direction but is perceived as spinning slowly in a counter-clockwise direction. The sampling capacity of the retina is thought to be determined by the peak number of ganglion cells, however, amacrine cells may also be involved. Indeed, AII amacrine cells form the limit of scotopic acuity, and there is considerable agreement between the maximum scotopic acuity and the peak density of AII cells, in the primate (Mills and Massey, 1999). Therefore, as the sampling rate of the retina must be greater than twice the highest frequency component in the signal (the Nyquist frequency) for detection without aliasing (Geisler and Hamilton, 1986), it follows that the absence of all Prdm13+ amacrine cells, of which there are many, may considerably decrease the retinal neural sampling density, and therefore increase the likelihood of aliasing artifacts. Importantly, this explanation is limited by the lack of documented amacrine cell types implicated in this phenomenon, and therefore it is unclear whether the amacrine subtypes affected in *Prdm13*^−∕−^ mice are specifically involved in the neural sampling of the retina. Whilst speculative, we believe this possibility warrants further investigation.

In summary, Watanabe et al. have comprehensively characterized the molecular features of amacrine cells whose specification is determined by Prdm13. Their findings indicate that Prdm13+ amacrine cells may negatively modulate visual sensitivities, an interesting hypothesis requiring further investigation. We speculate that an alternative explanation for these findings is that the reduction of Prdm13+ amacrine cells may have simply limited the sampling capacity of the retina, leading to aliasing artifacts. Further investigation of Prdm13+ amacrine cells and their role in OKR performance will be necessary to address this issue. Nevertheless, the recent work of Watanabe et al. confirms the crucial role for amacrine cells in retinal visual information processing, and provides essential insight into the complex nature of the retinal amacrine cell.

## Funding

NHMRC CJ Martin Fellowship (1072706) for MJ.

### Conflict of interest statement

The authors declare that the research was conducted in the absence of any commercial or financial relationships that could be construed as a potential conflict of interest.
